# A Study of Differences in Leisure Satisfaction of Leisure Activity Patterns for South Korean Adults

**DOI:** 10.3390/ijerph182010790

**Published:** 2021-10-14

**Authors:** Byoung-Wook Ahn, Won-Ick Song

**Affiliations:** 1 Department of Leisure Marine Sports, School of Marine-Sports, Seosan Campus, Hanseo University, Seosan-si 31962, Korea; bwahn75@hanseo.ac.kr; 2 Department of Sport Leisure Industry, Faculty of Art, Dangjin Campus, Sehan University, Dangjin-si 31746, Korea

**Keywords:** leisure satisfaction, leisure activity pattern, latent mean analysis, effect size

## Abstract

The purpose of this study was to analyse differences in leisure satisfaction among leisure activity participants according to the type of activity including differences by gender. The study subjects were 448 adult men and women who were participating in leisure activities in Seoul, Gyeonggi, Chungcheong, and Gangwon-do, Korea. Frequency analysis, confirmatory factor analysis, and latent mean analysis were applied to the data collected from the participants. First, the form, measurement, and intercept uniformity were verified to check that the study scale could be used equally with men and women. Second, it showed that leisure satisfaction was higher in sports activity of leisure activity patterns than hobbies, watching, socializing, tourism and games. It is concluded that infrastructure for sports should expand and that policy support is needed to increase leisure satisfaction in other leisure activities.

## 1. Introduction

The concept of well-being arose in the 1970s, related to health and quality of life, and spread worldwide [1]. In Korea, the five-day work week and the spread of recreational vehicles, which began on 1 July 2004, led to an increase in leisure time [2]. Outdoor Recreation Participation Report of the USA can be considered as an indicator of this situation. It is stated in this report that approximately half of the population in the USA participate in outdoor recreation activities, 6.1 million Americans are employed in outdoor recreation related jobs, and outdoor industry creates an economy of more than 640 billion [3]. In an effort to integrate the various perspectives on leisure, Murphy objected to the existing simple definitions of the concept of leisure, and proposed considering various aspects of leisure such as psychological, time classification, functional, social, and environmental [4]. Kelly defined leisure as based on subjective experience while acknowledging an integrated view of the concept and stated that it is easiest for individuals to select their own leisure activities because the associated feelings are specific to each individual [5]. Kelly reported the objective and psychological aspects of leisure activities and experiences of the phenomenon were integrated. Other researchers observed that individuals’ psychological traits determine what they consider to be leisure activities [6], but irrespective of the differing definitions and perceptions of leisure activities, they all bring benefits such as improvements in health, confidence, and social interaction [7].

Modern society supports a variety of creative leisure activities that give productive meaning and recharge individuals to cope with everyday life. Leisure activities can create positive effects by providing satisfaction not only in individual activities, but also in group activities [8]. Satisfaction develops as a result of the positive perception of leisure activities and is considered as an indicator of the level of satisfaction of an individual in meeting the requirements of that individual in experiential terms [9,10]. The fact that the demographic, socio-cultural, educational, psychological, and economic factors that affect leisure satisfaction vary between individuals shows that the concept can include a wide range of variables [11,12,13]. In an examination of differences in leisure satisfaction according to type of leisure activity, sports and travel offered more leisure satisfaction than did other types of activities that play an important role in the sub-variable of leisure satisfaction [14].

Leisure satisfaction is the essential function of leisure [15]. It refers to the subjective awareness of being happy or satisfied with a general leisure experience reflected as positive perceptions or emotions [16]. Contributors to leisure satisfaction include companionship during an activity characteristic, physical ability, cultivation of educational values, etc., and research confirms positive effects of participation in leisure activities on overall life satisfaction [2]. Schreyer and Driver [17] published a leisure benefit theory of integrative satisfaction. Additionally, Driver [18] established the concept and research method of BAL (Benefits Approach to Leisure). Leisure benefits are a subjective concept and relate to personal experiences [19]. This concept has been widely discussed and researched in the fields of physiology, psychology, sociology, and economics. Leisure benefits in these areas were examined as a subjective experience for each individual. These experiences help people to improve their mental and physical health, and to satisfy their physical and mental needs [20]. Leisure benefits are classified under three headings: physical, psychological, and social benefits [21]. Physical benefits refer to physical appearance preservation, energy gain, development of abilities for activities, regular resting, fatigue removal, and extra energy release. Psychological benefits include relief from life pressures, emotional relaxation, creative thinking, relaxation of mind and body, and enjoyment of life. Social benefits include the creation of new friendships and relationships, to be considered by other people, understanding the feelings of the people, and gaining the trust of others. Representative examples of this leisure benefit study include “Park usage, social milieu, and psychological benefits of park use reported by older urban park users from four ethnic groups”, “Benefits-based management of recreation services”, and “Climate change and recreation benefits in an Alpine national park” [22,23,24]. In modern society, leisure satisfaction is expanding from personal emotions to subjective benefit.

Most scholars who study social science use structural equation models (SEMs) to verify causal relationships [25]. The advantages of the structural equation are as follows. First, in SEMs, a common variable extracted using several measured variables is used as a latent variable, so the measurement error of the variable can be controlled; in other words, the structural equation model gives a more reliable estimate than the model based on only the measured variable because the SEM considers the measurement error. Second, it is easy to use parameters with SEMs. The characteristics of parameters are such that they must simultaneously play the role of independent and dependent variables in models. In the case of regression analysis, because one variable must play only one role, it is not easy to introduce and evaluate multiple parameters; using path rather than regression analysis allows for controlling parameters because using measurement variables rather than latent variables in the path analysis allows for properly controlling measurement errors. Third, SEMS make possible statistical evaluation of a theoretical model; researchers can evaluate how well the developed theoretical model fits the actual data and accept or modify the model as a valid model based on their findings [26]. Reviews of the SEM literature reveal rapidly increasing use of SEM procedures in recent decades, both in general and as they apply to tests for multi-group equivalence [27,28,29].

Given the above findings on different types of leisure satisfaction and how they vary depending on different leisure activities (hobbies, travel, viewing, sports, entertainment, etc.), a study was warranted that encompassed the different research constructs to verify leisure satisfaction according to the types of leisure activities and to use structural equation modeling to determine how well the theoretical model fit with actual data. The study aim was to identify satisfaction with various leisure activities and induce active participation in positive leisure activities.

## 2. Materials and Methods

### 2.1. Study Subject

The subjects of this study were adult men and women who lived in Seoul, Incheon, Gyeonggi, Chungcheong, and Gangwon-do, Korea, and who continuously participated in specific leisure activities. To verify the differences in leisure satisfaction according to type of leisure activities, allocation sampling was applied by region, and data of 448 out of 500 were used for actual analysis as shown in Table 1. The survey response rate was 89.6%. As for the sampling method, the random sampling method, quota sampling, was used. The study subjects were selected as adults who participated in leisure activities in each region for at least 3 months and 100 people were selected from each region. Due to constraints related to COVID-19, the study data were collected via non-face-to-face surveys, emails, and postal surveys. When face-to-face contact was possible, the researcher and assistant researcher sufficiently explained the purpose of the study in advance and obtained research consent, and then participants completed the self-report survey on their own.

### 2.2. Study Instrument

For the study research tool, the researchers used questions from Ahn, Yeo and Koo’s [30] Q methodological study on leisure satisfaction of participants in leisure activities and Ahn’s [31] analysis of equivalence and latent mean on leisure satisfaction of Korean adults, modified to suit this study’s purpose. Examples of leisure satisfaction items include “leisure activities are new experiences (self-development)”, “leisure activities help to change mood (stress solution)”, “building confidence through leisure activities (promotion of health)”, “leisure activities’ skills improve when participating (developing skills)”, and “participation in leisure activities understands each other (interpersonal relation)”. All questionnaires were answered on a scale from ‘strongly disagree (1 point)’ to ‘strongly agree (5 points)’ using a 5-point Likert scale.

### 2.3. Validity and Reliability

Confirmatory factor analysis (CFA) is used to confirm underlying factor dimensions in testing hypotheses based on the knowledge of the researcher [32]. To verify the construct validity of this study, the fitness index was used instead of χ^2^ as an evaluation index; this was because the content of the zero hypothesis of the χ^2^ verification is strict, the χ^2^ is sensitive to sample size, and models consequently tend to be rejected too easily [33]. Among the popular goodness-of-fit indices, the Tucker-Lewis index (TLI), comparative fit index (CFI), and root mean square error of approximation (RMSEA) are the most widely used because they are not affected by sample size; additionally, the TLI and RMSEA in particular, consider not only the explanatory power of the model but also its simplicity [27]. The CFA results for this study were as follows: leisure satisfaction χ^2^/df = 2.729, TLI = 0.913, CFI 0.929, and RMSEA = 0.079; all values indicated satisfactory goodness of model fit as shown in Table 2. Additionally, Cronbach’s α was calculated to verify the reliability of this study; an index greater than 0.6 indicates reliability [34]. The Cronbach’s alphas for the components of leisure satisfaction that we studied were 0.751, 0.723, 0.811, 0.806 and 0.792, and the reliability coefficient for overall leisure satisfaction was 0.891.

### 2.4. Data Collation and Processing

This quantitative study entailed collecting participant data by conducting a questionnaire survey. It analyzed the collected data using SPSS 21.0 and AMOS 18.0. Demographic characteristics of the study subjects were subjected to frequency analysis, and CFA verified the validity of the measurement tool. Cronbach’s α confirmed internal consistency as well. Among the structural equation modeling methods, equivalence verification (configural, matric, and scalar invariance), latent mean analysis, and effect size analysis were conducted.

### 2.5. Ethics Statement

All of the study procedures were reviewed and approved by the Hanseo University Department of Sports Research Institutional Review Board and conducted according to the principles expressed in the Declaration of Helsinki. After being provided with explanations of the purposes and length of this research study, all participants provided consent to participate; they understood that they could refuse to participate in this research study at any time. The participants agreed to allow researchers to use their personal information, which was obtained from questionnaires, for the aim of this study.

## 3. Results

### 3.1. Equivalence

#### 3.1.1. Configural Invariance

To verify the configural invariance of the measurement model, the leisure satisfaction variable was compared between the men and the women in the study sample (women: χ^2^ = 170.120, χ^2^/df 2.849, TLI = 0.910, CFI = 0.928, RMSEA = 0.075; men: χ^2^ = 210.682, χ^2^/df = 2.695, TLI = 0.918, CFI = 0.920, RMSEA = 0.074). The fit of the base model, which allowed correlations between all latent variables and free measurement of the parameters, was found to be satisfactory for both groups. According to Browne and Cudeck, RMSEA less than 0.05 indicates adequate fit, good fit ranges between 0.05 and 0.08, and fit is poor if RMSEA is more than 0.10 [35]. TLI and CFI appear differently depending on the continuum from 1 to 0, but values for both above 0.90 can be said to reflect model suitability [36,37]. TLI = 0.898, CFI = 0.928, and RMSEA = 0.026, all indicate that the model was suitable for the data. In this study, after morphological identity was verified according to gender, the fitness indices were it.

#### 3.1.2. Matric Invariance

As matric invariance was established, the next step, which assumed that the factor coefficients of the two groups were the same, was to verify the measurement identity to determine whether the model fit deteriorated. The χ^2^ difference test was conducted to determine whether the two models would show a significant difference by comparing the model with the same factor coefficients (Model 2) and the base model without any restrictions (Model 1). As the difference was 90 and the degree of freedom between models was 55, the difference was not significant at α = 0.05, and thus, the measurement identity was secured (TLI = 0.889, CFI = 0.881, RMSEA = 0.026). It is judged that the scale items could be applied equally to men and women as shown in Table 3.

#### 3.1.3. Scalar Invariance

As the measurement identity was established, the intercept identity was verified as well. Scalar invariance was verified by confirming the fit between the measurement identity model in which the factor coefficients were the same between groups, and the intercept identity model in which the identification constraints were applied to the intercepts of each measurement variable. In the verification findings, the χ^2^ difference was 170 and the degree of freedom between the models was 80, which was not significant at α = 0.05; as such, there was no difference between the two models, and the intercept identity was verified as sown in Table 4.

### 3.2. Latent Mean Analysis and Effect Size

As latent means cannot be directly estimated, it is necessary to measure the latent mean of the measurement group by assuming it as a latent mean in the comparison group. That is, the potential average of the different leisure activity groups (hobbies, watching, socializing, tourism, games) was assumed to be 0, and the potential average of the sports group was measured and compared. According to the criterion suggested by Cohen, *d* = 0.2 is a small difference, *d* = 0.5 is moderate, and >0.8 is large [38,39]. Table 5 presents the latent mean analysis findings for self-development (0.061), stress relief (0.190), health promotion (0.295), skill development (0.212), and interpersonal relations enhancement (0.248) as aspects of satisfaction during leisure activities; differences were significant at *p* < 0.001. The table results indicate that survey respondents in this study reported greater leisure satisfaction from participating in sports leisure activities than from participating in hobbies, watching, socializing, tourism, or games. In terms of effect size, Cohen’s *d* was highest for skill development, followed by health promotion, stress relief, and interpersonal relations enhancement. However, there were no significant differences in self-development satisfaction by leisure activity.

## 4. Discussion

The aim of this study was to investigate differences between men and women in leisure satisfaction. This was obtained from their participation in leisure activities by analyzing latent means for each type of activity. The objective was to elucidate subjective perceptions of satisfaction by evaluating whether the measurement tools used in this study could be used irrespective of gender and then verifying the relationships between the variables. The following is a discussion based on comparing the results of this study with previous studies.

First, the configural, matric, and scalar invariable was verified through the latent mean analysis to determine whether the measurement tool of the study can be used regardless of gender. It was confirmed that the study variables were effective for measurement among both men and women. Park and Cheon considered it necessary to develop and verify a variety of scales for different leisure activities [40]. Kim argued that individual researchers should validate the scales they use rather than just using scales developed by previous researchers [41]. In addition, researchers, to date, have mainly focused on the relationships among research variables but have overlooked the most important variables, the demographic statistics. Moreover, researchers did not establish whether their measurement tools could be used equally between genders. In this study, it was possible to more accurately identify differences in leisure satisfaction both by type of leisure activity and according to the gender.

Second, differences in latent means were identified by gender and by activity type. By gender, men were more satisfied with their leisure activities than were women in terms of skill development, health promotion, stress solution, and interpersonal relations enhancement; Cohen’s *d* of 0.5 indicated a moderate effect size for stress solution, one of the many established benefits of participating in leisure activities. Kim and Kim found that fencing participants gained emotional satisfaction from fencing through learning how to overcome difficulties and solve problems, which also boosted their confidence [42]. Eifert, Hall, Smith, and Wideman determined that emotional satisfaction increases when physical function develops and achievements are reached, and the emotional satisfaction has the positive impact of increasing focus on exercise [43]. Ahn, as well, identified that participating in leisure activities relieves stress and contributes to self-improvement and self-development, which ultimately benefits societies [44].

The effect sizes for skill development and health promotion were greater than 0.7 for satisfaction with sports activities; it is believed because of the physical health and confidence that result from the skills development, and the improved physical health through exercise. Park said that the more aware he is of his own health, the more satisfied he is with his leisure time [45]. Kim, Kim, and Moon, and Lee and Moon identified a causal relationship between health beliefs and leisure satisfaction [46,47]. Roh found that unmarried women highly valued their physical leisure activities as part of their high awareness of health maintenance [48].

The effect size for interpersonal development was slightly lower at 0.4. The start of the five-day work week afforded more time for participating in leisure activities, and many activities, both passive and active, entail interacting with a variety of different people. In addition to sports, meeting new people increases satisfaction with participating in hobbies, socializing, and games; meanwhile, sports are a leisure activity that can be engaged in passively by just watching, and this can be more enjoyable to watch with a large number of people than alone. Kim and Kim [49], Lee [50], and McFarlane [51] identified importance and centrality as components of the emotional dimension of specialization that influenced educational and social satisfaction, and Kim and Kim’s [52] study participants who enjoyed cultural and artistic activities reported that they preferred activities they could enjoy with others. Kellison, Kim, and Magnusen found that millennial employees had influenced leisure satisfaction in each generation [53]. For workers, leisure life with family or clubs had significant positive effects on leisure satisfaction, and millennials preferred leisure with friends to being alone. Petrick and Backman found that leisure satisfaction and the need for interpersonal relationships were affected by social friendliness and interactions, and Shin and Kim found that unmarried women spent much time during their weekends on interpersonal social activities such as meeting friends and colleagues [54,55]. Eifert, Hall, and Wideman also found that women’s participation in physical activity had high positive effects on their interpersonal satisfaction [43].

In this study, latent mean analysis was conducted to verify the differences in the research variables. Quantitative researchers have overlooked the demographic variable of gender and have only studied causal relationships. However, findings for sports activities, in particular, differ depending on gender, and as such, it seems necessary to study demographic variables such as gender. It was established in this study that the measurement tool selected could be used regardless of gender. The study has contributed practically and academically through its more accurate research results on leisure satisfaction by both leisure activity and gender.

## 5. Practical Application

For policymakers and leisure managers, the current study has some pragmatic implications, such as for how to improve leisure satisfaction in leisure activity participants. The data from this study are expected to be used as policy data for establishing a proper leisure culture by examining the actual conditions of leisure and types of leisure activities in Korea. As selecting and participating in leisure activities is centered on subjective and internal individual traits, subjective and conscious values could be influences on the types of activities people participate in; future scholars could conduct research in connection with other disciplines (cultural anthropology, sociology, psychology, etc.). In addition, it is expected that further research on leisure culture and leisure satisfaction could identify additional social and psychological factors that affect satisfaction with various leisure activities.

## 6. Limitations and Future Research

There are some limitations in the current study. Because the study was limited to participants in leisure activities in Seoul, Gyeonggi, Chungcheong, and Gangwon-do, the findings cannot be generalized to South Korea nationwide. Separately, leisure satisfaction is a personal feeling, and satisfaction characteristics according to each leisure activity type need to be approached through a qualitative study.

## 7. Conclusions

The purpose of this study was to analyze the differences in leisure satisfaction among leisure activity participants according to the types of leisure activities. The subjects of the study were adult men and women participating in leisure activities in Seoul, Gyeonggi, Chungcheong, and Gangwon-do, Korea. The data were processed using frequency analysis, confirmatory factor analysis, equivalence and latent mean analysis, and the following findings resulted. Firstly, form uniformity, measurement uniformity, and intercept uniformity were verified to confirm that the scale could apply to both men and women for any type of leisure activity. Second, sports activities were more satisfying to the men in this study than were hobbies, watching, socializing, traveling, and game activities after measurement errors were controlled for. Given that sports leisure activities showed such high leisure satisfaction in this study, it is necessary to expand the infrastructure for these activities nationwide. In addition, there is a need to develop and apply programs that can increase leisure satisfaction with other leisure activities.

## Figures and Tables

**Table 1:** Description of participants (*N* = 488).

Item		*N*	%	Item		*N*	%
Gender	Male	218	48.7	Area	Seoul	99	20.4
	Female	230	51.3		Incheon	97	20.0
Age	20s	187	41.7		Gyeonggi	98	20.2
	30s	107	23.9		Chungcheong	96	19.8
	40s	73	16.3		Gangwon	95	19.6
	50s and over	81	18.1	Participate Period	Under 1 year	140	31.3
Leisure Activity	Sports	143	31.9		1–3 year under	126	28.1
	Hobbies	65	14.5		3–5 year under	66	14.7
	Viewing	108	24.1		5 year over	116	25.9
	Social	50	11.2	Participate Frequency	1 time	183	40.8
	Tourism	25	5.6		2 times	128	28.6
	Entertainment	57	12.7		3 times over	137	30.6

**Table 2:** Fit indices for confirmatory factor and reliability analysis.

Item	Estimate	S.R.	S.E.	C.R.	Cronbach’s α
Ls3	1.000	0.435			0.751
Ls2	1.194	0.633	0.114	10.473 ***	
Ls1	1.201	0.583	0.118	10.217 ***	
Ls7	1.000	0.649			0.723
Ls6	1.144	0.856	0.059	19.314 ***	
Ls5	1.030	0.343	0.106	9.690 ***	
Ls4	0.837	0.668	0.047	17.643 ***	
Ls10	1.000	0.706			0.811
Ls9	1.177	0.871	0.051	23.167 ***	
Ls8	1.082	0.728	0.051	21.013 ***	
Ls14	1.000	0.586			0.806
Ls13	1.288	0.717	0.088	14.609 ***	
Ls12	1.119	0.547	0.089	12.632 ***	
Ls11	0.992	0.572	0.075	13.176 ***	
Ls17	1.000	0.681			0.792
Ls16	1.119	0.652	0.080	13.927 ***	
Ls15	0.729	0.576	0.055	13.342 ***	
Model fit: χ^2^/df = 2.729, TLI = 0.913, CFI 0.929, and RMSEA = 0.079					

Note: S.E. = standard error; C.R. = critical ratio; *** *p* < 0.001.

**Table 3:** Results for goodness-of-fit for identity verification.

Model	χ^2^	df	TLI	CFI	RMSEA
Model 1: Configural invariance	857.982	510	0.898	0.928	0.026
Model 2: Matric invariance	948.471	565	0.889	0.881	0.026
Model 3: Scalar invariance	1208.846	645	0.870	0.883	0.029
Model 4: Factor variance	1253.310	670	0.874	0.899	0.029

**Table 4:** χ^2^ results for latent mean analysis.

Model	Δx	Δdf	Adoption
Matric invariance (Model 1 vs. Model 2)	90.489	55	Accept
Scalar invariance (Model 2 vs. Model 3)	260.345	80	Accept
Factor variance identity (Model 3 vs. Model 4)	44.464	25	Accept

**Table 5:** Latent means for leisure satisfaction by leisure activity.

	Sport		Hobby, Viewing, Social, Tour, Game		Effect Size	Total Mean
	Latent M	M	Latent M	M		
Self-development	0	3.619	−0.061	3.593	0.290	3.583
Stress solution	0	4.089	−0.190 ***	3.936	0.549	3.973
Health promotion	0	3.911	−0.295 ***	3.631	0.735	3.717
Skill development	0	3.649	−0.212 ***	3.415	0.837	3.488
Interpersonal relations	0	3.262	−0.248 ***	3.058	0.467	3.097

*** *p* < 0.001.

## Data Availability

The Study did not report any data.

## References

[1] Ahn B.W., Kim D.K., Seol S.H., Lee S.J. (2020). A study of leisure industry development in South Korea: Structural relationship between leisure facilitation, recreation specialization and wellness. J. Adv. Res. Dyn. Control Syst..

[2] Ahn B.W., Chon T.J. (2018). Verification of relationships of serious leisure, leisure facilitator and psychological happiness among leisure sports participants in South Korea. Asia Life Sci. Suppl..

[3] Outdoor Industry Association (2017). The Outdoor Recreation Economy.

[4] Murphy J.F. (1981). Concepts of Leisure.

[5] Kelly J.R. (1900). Leisure.

[6] Sung Y., Ko D., Joon J. (1996). Psychological meaning of leisure. Korean J. Ind. Organ. Psychol..

[7] Lee C. (2012). Exploring the relationship between Leisure and self-management. J. Leis. Wellness.

[8] Hur N. (2015). The relationship between leisure satisfaction and self-realization according to the type of university students` leisure activities. Korean Soc. Leis. Recreat..

[9] Yasarturk F. (2019). Analysis of the relationship between the academic self-efficacy and leisure satisfaction levels of university students. J. Educ. Train. Stud..

[10] Lloyd K.M., Auld C.J. (2002). The role of leisure in determining quality of life: Issues of content and measurement. Soc. Indic. Res..

[11] Beard J.G., Ragheb M.G. (1980). Measuring leisure satisfaction. J. Leis. Res..

[12] Rojek C. (2013). Capitalism and Leisure Theory (Routledge Revivals).

[13] Esentaş M., Güzel P., Yıldız K., Çokşen M. (2018). The role of the quality of life of the participation to the time leisure time activities in nursing room. J. Phys. Educ. Sports Scie..

[14] Siegenthaler K.L., O'Dell I. (2000). Leisure attitude, leisure satisfaction, and perceived freedom in leisure within family dyads. Leis. Sci..

[15] Yaşartürk F., Akyüz H., Karataş İ. (2017). Examination of university students’ levels of leisure boredom perception and life satisfaction towards recreative activities. Int. J. Cult. Soc. Stud..

[16] Pohl S.L., Borrie W.T., Patterson M.E. (2000). Women, wilderness, and everyday life: A documentation of the connection between wilderness recreation and women’s everyday lives. J. Leis. Res..

[17] Schreyer R., Driver B., Jackson E., Burtob T. (1989). The benefits of leisure. Understanding Leisure and Recreation; Mapping the Past, Charting the Future.

[18] Driver B., Hamilton Smith E. (1990). The North American experience in measuring the benefits of leisure. Proceedings, National Workshop on Measurement of Recreation Benefits.

[19] Kao C.H. (1995). A three-factor model of leisure benefits. J. Outdoor Recreat. Study.

[20] Chen Z.Y. (2001). The Study of Elementary Teachers’ Leisure Participation, Experience in Leisure Benefits, and Work Satisfaction in Taipei County.

[21] Hung H.J. (2012). A study on leisure benefits breaking through leisure activities. J. Natl. Taiwan Norm. Univ..

[22] Tinsley H.F., Croskets C.F. (2002). Pars usage, Social milieu and psychological benefits of pars use reported by older urban park users form four ethnic groups. Leis. Sci..

[23] Allen L.A. (1996). Primer: Benefit-based management of recreation services. Park Recreat..

[24] Robert B.R., John B.L. (2005). Climate change and recreation benefits in an Alpine National Park. J. Leis. Res..

[25] Kim J., Kim M., Hong S. (2009). Writing Thesis Using Structural Equations.

[26] Austin J.T., Calder´on R.F. (1996). Theoretical and technical contributions to structural equation modeling: An updated annotated bibliography. Struct. Equ. Model..

[27] Hershberger S.L. (2003). The growth of structural equation modelling: 1994–2001. Struct. Equ. Model..

[28] Tremblay P.F., Gardner R.C. (1996). On the growth of structural equation modeling in psychological journals. Struct. Equ. Model..

[29] Vandenberg R.J., Lance C.E. (2000). A review and synthesis of the measurement equivalence literature: Suggestions, practices, and recommendations for organizational research. Organ. Res. Methods.

[30] Ahn B., Yeo I., Ko M. (2009). A Q-methodology study on the leisure satisfaction patterns for leisure activity participants. Korean J. Phys. Educ..

[31] Ahn B. (2016). Analysis of Equivalence and Latent Mean on Leisure Satisfaction of Korean Adults. Information.

[32] Kim K. (2011). Amos 18.0 Structural Equation Model.

[33] Hong S. (2000). The criteria for selecting appropriate fit indices in structural equation modeling and their rationales. Korean J. Clin. Psychol..

[34] Sung T. (2006). Theory and Practice of Item Creation and Analysis.

[35] Browne M.W., Cudeck R., Bollen K.A., Long J.S. (1993). Alternative ways of assessing model fit. Testing Structural Equation Models.

[36] Bentler P.M. (1990). Comparative fit indexes in structural models. Psychol. Bull..

[37] Tucker L.R., Lewis C. (1973). A reliability coefficient for maximum likelihood factor analysis. Psychometrika.

[38] Cohen J.O. (1988). Statistical Power Analysis for the Behavioral Sciences.

[39] Hu L.T., Bentler P. (1999). Cut-off criteria for fit indexes in covariance structure Analysis; Conventional criteria versus new alternatives. Struct. Equ. Model..

[40] Park S., Chon T. (2010). A comparative study of latent means of leisure satisfaction between college sport and no-sport club participants. J. Leis. Recreat. Stud..

[41] Kim E. (2008). Multi-group confirmatory factor analysis and latent mean analysis of the TEOSQ scale-II. J. Sport Leis. Stud..

[42] Kim E., Kim Y. (2020). The effect of leisure engagement of Fencing club participants on leisure satisfaction. Korea J. Sports Sci..

[43] Eifert E.K., Hall M., Smith P.H., Wideman L. (2019). Quality of life as a mediator of leisure activity and perceived health among older women. J. Women Aging.

[44] Ahn B. (2017). The structural relationship between serious leisure, leisure support, leisure satisfaction and psychological happiness among leisure activity participants. Korean J. Phys. Educ..

[45] Park S. (2020). The relationship among health-perception, exercise enjoyment and leisure satisfaction for College Students attended physical education. Korean J. Sports Sci..

[46] Kim J., Kim K., Moon H. (2018). The effects of health belief on health promotion education and leisure satisfaction among adolescents on the growth phase. Korean J. Growth Dev..

[47] Lee K., Lee S. (2011). Effects of health perception and health promotion behavior on leisure satisfaction among the Middle-Aged participants in physical activities. Korean J. Sports Sci..

[48] Roh Y. (2017). Awareness of Aging in Unmarried Middle-Aged Women. Unpubished Ph.D. Thesis.

[49] Kim H., Kim N. (2012). Analysis on cyclists’ segmentation and cycling environment preferences according to recreation specialization. J. Tour. Sci..

[50] Lee S. (2015). The Influence of Recreation Specialization of Horse Riders on Leisure Commitment and Satisfaction. Unpublised Master’s Thesis.

[51] McFarlane B. (1994). Specialization and motivations of birdwatchers. Wildl. Soci. Bull..

[52] Kim E., Kim Y. (2020). Analysis of the relationship between leisure involvement depending on the types of leisure activities and leisure satisfaction of the Korean. J. Korean Soci. Rhythm. Exerc..

[53] Kim S., Kim N. (2020). Comparison of the impacts of leisure environment on leisure satisfaction in Baby Boom Generation and millenial generation: Focused on the leisure sharer. J. Tour. Stud..

[54] Petrick J.F., Backman S.J. (2002). An Examination of the Determinants of Golf Travelers’ Satisfaction. J. Travel Res..

[55] Shin W., Kim J. (2014). The leisure culture of unmarried working women in Korea. J. Korea Cult. Ind..

